# New Metabolites from Endophytic Fungus *Chaetomium globosum* CDW7

**DOI:** 10.3390/molecules23112873

**Published:** 2018-11-04

**Authors:** Wei Yan, Ling-Ling Cao, Yang-Yang Zhang, Ran Zhao, Shuang-Shuang Zhao, Babar Khan, Yong-Hao Ye

**Affiliations:** 1 State & Local Joint Engineering Research Center of Green Pesticide Invention and Application, College of How Plant Protection, Nanjing Agricultural University, Nanjing 210095, China; yanwei@njau.edu.cn (W.Y.); 2015202050@njau.edu.cn (L.-L.C.); zyy2857@163.com (Y.-Y.Z.); 2017102130@njau.edu.cn (R.Z.); 2017202055@njau.edu.cn (S.-S.Z.); babarkhan.uaf@gmail.com (B.K.); 2 Key Laboratory of Integrated Management of Crop Diseases and Pests, Ministry of Education, Nanjing 210095, China

**Keywords:** *Chaetomium globosum*, isocoumarin derivatives, indole alkaloid, antifungal activity

## Abstract

Five metabolites including two new ones, prochaetoviridin A (**1**) and chaetoindolin A (**2**), were isolated from the endophytic fungus *Chaetomium globosum* CDW7. Compounds **1** and **2** were characterized as an isocoumarin and an indole alkaloid derivative, respectively, with their structures elucidated by comprehensive spectroscopic analyses including high-resolution electrospray ionization mass spectrometry (HR-ESI-MS), NMR, and circular dichroism (CD) comparison. Compounds **3**–**5** were identified as chaetoviridin A, chaetoglobosin R, and chaetoglobosin T, respectively. Chaetoviridin A (**3**) exhibited antifungal activity against *Sclerotinia sclerotiorum* with an EC_50_ value of 1.97 μg/mL. In vivo test showed that **3** displayed a protective efficacy of 64.3% against rape *Sclerotinia* rot at the dosage of 200 μg/mL, comparable to that of carbendazim (69.2%).

## 1. Introduction

Plant diseases caused by phytopathogenic fungi are one of the major problems contributing to crop loss. Over several decades, synthetic fungicides have been primarily developed to prevent and control plant diseases. However, the global trend appears to be shifting towards a reduced use of fungicides, and hence there is an urgent need for safer eco-friendly alternatives to treat plant diseases. Natural products, with their wide spectrum of bioactivities and environmentally friendly attributes, are the most promising source of lead molecules for agricultural chemicals [1,2].

Endophytic fungi are considered as prolific producers of natural products with structural and biological diversity [3,4]. *Chaetomium globosum* is a well-known member of the *Chaetomiaceae* family, which commonly resides on plants, soil, straw, and dung [5,6]. A large number of structurally diverse metabolites, such as chaetoglobosins, azaphilones, xanthones, and steroids, have been characterized from *C. globosum* species. These structures display a wide range of biological activities including anticancer, antimicrobial, immunosuppressive, and antioxidant [6,7,8,9,10,11,12].

**Figure 1:**
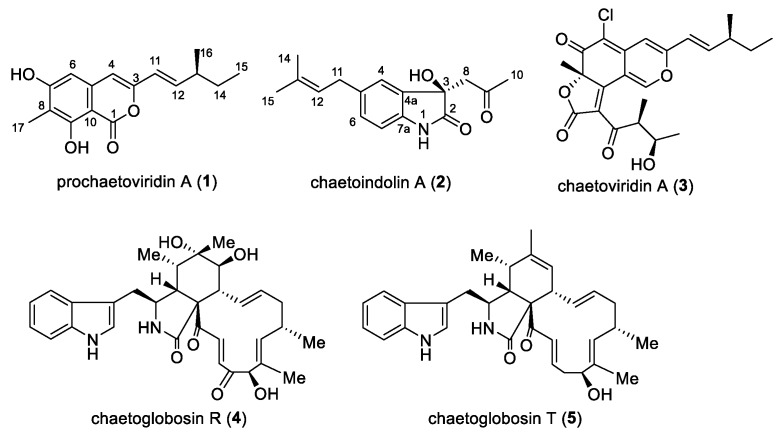
Structures of compounds **1**–**5**.

Previously, we reported that *C. globosum* CDW7, an endophyte from *Ginkgo biloba*, exhibited strong inhibitory activity against plant pathogenic fungi in vitro. To explore the associated substance regarding its antifungal activities, flavipin, chaetoglobosins A and D were isolated using the bioassay-guided method [13,14,15]. During our ongoing search for new bioactive metabolites, one new isocoumarin derivative, prochaetoviridin A (**1**), and one new indole alkaloid, chaetoindolin A (**2**), together with chaetoviridin A (**3**), chaetoglobosins R and T (**4** and **5**) [16,17], were isolated from this fungus (Figure 1). Chaetoviridin A has been reported to be antifungal against some phytopathogens such as *Rhizoctonia solani*, *Magnaporthe grisea* and *Pythium ultimum* [18,19]. To the best of our knowledge, this is the first study of its activity against *Sclerotinia sclerotiorum* both in vitro and in vivo.

## 2. Results and Discussion

### 2.1. Structure Elucidation

**Figure 2:**
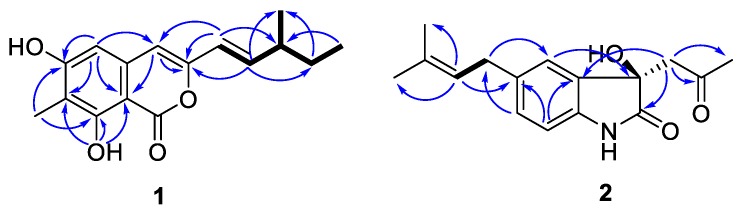
Key ^1^H-^1^H COSY (bold) and HMBC (solid arrows, blue) correlations of compounds **1** and **2**.

Prochaetoviridin A (**1**) was obtained as a light-yellow powder. Its molecular formula, C_16_H_18_O_4_, with eight degrees of unsaturation, was determined by high-resolution electrospray ionization mass spectrometry (HR-ESI-MS) (*m*/*z* 297.1099 ([M + Na]^+^; calcd. 297.1097). The ^1^H NMR spectrum of **1** indicated the presence of three methyl groups (one singlet, one doublet, and one triplet), one methylene and one methine proton, one trans-olefinic group (*J* = 15.6 Hz), two uncoupled olefinic or aromatic protons, and one hydroxyl group (δ_H_ 11.42). The ^13^C NMR spectrum revealed the existence of one lactone group (δ_C_ 166.2) and one benzene ring (C-atoms ranging from δ_C_ 100.0 to 161.3). The ^1^H-^1^H COSY spectrum suggested the presence of a 3-methyl-1-pentenyl group. The HMBC correlations from H-6 (δ_H_ 6.29) to C-8, C-4, and C-10, from H-17 (δ_H_ 2.17) to C-7, C-8, and C-9, and from H-4 (δ_H_ 6.15) to C-10 constructed the core structure of **1**. The 3-methyl-1-pentenyl side chain was at the 3-position of the core ring as elucidated by the HMBC correlation from H-11 (δ_H_ 5.95) to C-3 and C-4 (Table 1). Thus, the whole structure was pieced together as shown in Figure 2. The stereochemistry of C-13 in the side chain is usually established by chromium trioxide oxidation [16,20], but we were unable do this experiment due to sample scarcity. Since compound **1** was closely related to the biosynthesis of chaetoviridin A (**3**), its absolute configuration was proposed as 13S, the same as the side chain of **3**.

**Figure 3:**
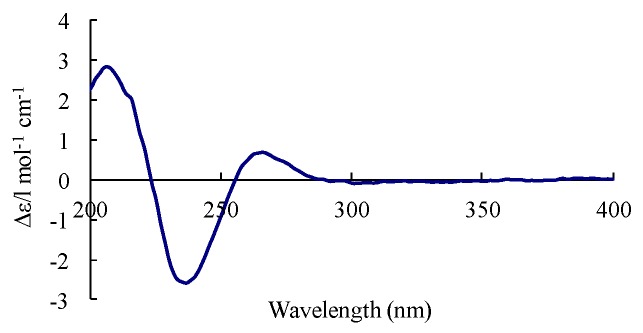
Circular dichroism (CD) spectrum of compound **2**.

Chaetoindolin A (**2**) was isolated as a colorless amorphous solid with the molecular formula C_16_H_19_NO_3_ as evidenced by HR-ESI-MS. The ^1^H and ^13^C spectra revealed three aromatic methines (H-4 (δ_H_ 7.15, s), H-6 (δ_H_ 7.05, d, *J* = 7.8 Hz), and H-7 (δ_H_ 6.77, d, *J* = 7.3 Hz)) and three quaternary aromatic carbons, suggesting the presence of a 1,2,4-trisubstituted benzene ring. This was verified by HMBC correlations from H-4 to C-6 and C-7a, H-7 to C-4a and C-5, and H-6 to C-4 and C-7a. An isoprenyl unit was deduced by ^1^H-^1^H COSY correlation between H-11 (δ_H_ 3.29, d, *J* = 7.3 Hz) and H-12 (δ_H_ 5.26, d, *J* = 7.3 Hz) and HMBC correlations from H-12 to C-14 and C-15, and was indicated to be attached at C-5 mainly by the HMBC cross peaks for H-6/C-11 and H-11/C-4 (Table 2). Considering the molecular formula and the chemical shift of C-3 (δ_C_ 74.8), a hydroxyl group was supposed to be at C-3, indicating the presence of a 3-hydroxyoxindole ring. The HMBC correlations from H-8 (δ_H_ 2.98 and δ_H_ 3.16) to C-10 and H-10 (δ_H_ 2.17) to C-8 and C-9 led to the elucidation of a 2-oxopropyl group, which was placed at C-3 by HMBC cross peaks for H-8/C-4a and H-8/C-2. The absolute configuration of **2** was determined by comparison of its circular dichroism (CD) spectrum with those of 3-hydroxyxoindole derivatives [21,22]. Compound **2** had a weak positive Cotton effect at 264 nm, a negative one at 238 nm, and a positive one at 215 nm (Figure 3), which resembled those of (*R*)-convolutamydine E [22]. Thus, we established the 3*R* configuration of **2**.

The structures of the other known compounds, chaetoviridin A (**3**), chaetoglobosin R (**4**), and chaetoglobosin T (**5**) were identified on the basis of their MS, ^1^H, and ^13^C NMR data by comparison with the data reported previously in the literature [16,17].

### 2.2. Biological Activity

**Figure 4:**
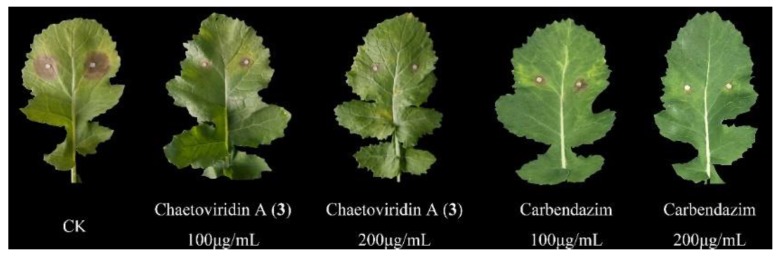
Effects of chaetoviridin A (**3**) against *S. sclerotiorum*-infected cole leaves. CK, control check (5% DMSO without compounds).

All isolated compounds were evaluated for their antifungal activities against pathogenic fungi at the concentration of 20 μg/mL. Prochaetoviridin A (**1**) showed moderate antifungal activity with inhibition rates ranging from 13.7% to 39.0%. Chaetoviridin A (**3**) was active against *S. sclerotiorum*, *Botrytis cinerea*, *Fusarium graminearum*, *Phytophthora capsici* and *F. moniliforme* with inhibition rates of 97.8%, 69.1%, 77.0%, 60.7%, and 59.2%, respectively. Other compounds (**2**, **4** and **5**) displayed no obvious effect (Table 3). The EC_50_ value of **3** against *S. sclerotiorum* was further determined as 1.97 μg/mL, compared to that of positive control (carbendazim, 0.17 μg/mL). In vivo test revealed that **3** could successfully inhibit disease development in *S. sclerotiorum*-infected rape with 45.2% and 64.3% protective efficiency and dosages of 100 and 200 μg/mL, respectively, which is comparable to those of carbendazim (44.6% and 69.2%) (Figure 4, Table 4).

## 3. Materials and Methods

### 3.1. General Experimental Procedures

The UV spectra were obtained from a Hitachi U-3000 spectrophotometer (Hitachi, Tokyo, Japan). Optical rotations were measured on a Rudolph Autopol III automatic polarimeter (Rudolph Research Analytical, Hackettstown, NJ, USA). CD spectra were acquired on a JASCO-810 spectropolarimeter (JASCO, Easton, MD, USA). NMR spectra were obtained using a Bruker DRX-600 NMR spectrometer (Bruker, Fällanden, Switzerland) at room temperature with TMS (tetramethylsilane) or solvent signals as calibration. High-resolution electrospray ionization mass spectrometry (HR-ESI-MS) results were recorded on an Agilent 6210 TOF LC-MS spectrometer (Agilent Technologies, Santa Clara, CA, USA). Silica gel (200–300 mesh) for column chromatography (CC) was purchased from Qingdao Marine Chemical Factory, Qingdao, China. Sephadex LH-20 was produced by Pharmacia Biotech, Uppsala, Sweden. Semi-preparative HPLC purification was carried out on a Kromasil 100-5-C18 column (5 μM, 250 × 10 mm, AkzoNobel, Shanghai, China). All chemicals used in the study were of analytical or HPLC grade.

### 3.2. The Source of Strains

*C. globosum* CDW7 and all tested plant pathogens were supplied and stored by the Laboratory of Natural Products and Pesticide Chemistry, Nanjing Agricultural University. The strains were cultivated in potato dextrose agar (PDA) at 25 °C after retrieval from the storage tube.

### 3.3. Fermentation, Extraction, and Isolation

Strain CDW7 was incubated on PDA at 25 °C for 5 days. Then, the mycelial agar plugs were transferred from the edge of the cultures to 1000 mL Erlenmeyer flasks containing 400 mL of Czapek’s medium (30 g sucrose, 1 g yeast extract, 3 g NaNO_3_, 0.5 g MgSO_4_·7H_2_O, 10 mg FeSO_4_·7H_2_O, 1 g K_2_HPO_4_, 0.5 g KCl, in a final volume of 1 L water), which was continuously shaken (150 rpm) for 10 days. The broth culture (40 L) was filtered through muslin cloth and extracted with ethyl acetate (EtOAc) three times to give the crude extract (60 g). The crude extract was subjected to silica gel column chromatography eluted stepwise with CH_2_Cl_2_–MeOH (100:0, 100:1, 100:2, 100:4, 100:8, and 0:100) as the mobile phase to afford six fractions, Fr1–Fr6. Fr2 was fractionated by CC over silica gel (EtOAc/petroleum, *v*/*v*, 100:0–0:100) to give five fractions (Fr2.1–Fr2.5). Fr2.3 was further separated on a Sephadex LH-20 column eluted with MeOH to yield compound **1** (1.8 mg). Fr4 was subjected to a Sephadex LH-20 column eluted with MeOH several times and separated by semi-preparative HPLC (MeOH/H_2_O, 75:25) to give **2** (2.4 mg, R_t_ = 17.5 min). Fr2.2 was separated by CC Sephadex LH-20 and purified by semi-preparative HPLC (MeOH/H_2_O, 85:15) to give **3** (27 mg, R_t_ = 25.6 min). Fr4.4 was subjected to silica gel and Sephadex LH-20 CC to afford **4** (5.5 mg) and **5** (7.2 mg).

*Prochaetoviridin A* (**1**): light yellow powder, [α${]}_{D}^{20}$ 2.5 (c 0.25, MeOH); UV (MeOH) λmax (log ε) 252 (2.9), 261 (3.1) nm; CD (MeOH) λmax (∆ε) 228 (+0.7), 253 (+1.7), 259 (+1.4); HR-ESI-MS *m*/*z* 297.1099 [M + Na]^+^ (cacld. C_16_H_18_O_4_Na, 297.1097). ^1^H and ^13^C NMR spectroscopic data, see Table 1.

*Chaetoindolin A* (**2**): colorless amorphous solid, [α${]}_{D}^{20}$ 1.8 (c 0.50, MeOH); UV (MeOH) λmax (log ε) 202 (2.0), 259 (0.2) nm; CD (MeOH) λmax (∆ε) 215 (+4.3), 238 (−3.2), 264 (+1.1); HR-ESI-MS *m*/*z* 296.1254 [M + Na]^+^ (cacld. C_16_H_19_NO_3_Na, 296.1257). ^1^H and ^13^C NMR spectroscopic data, see Table 2.

### 3.4. Antifungal Assays

The antifungal tests were conducted according to the protocols described in previous literature [15].

## 4. Conclusions

Rape *Sclerotinia* rot (RSR) caused by *S. sclerotiorum* seriously affects the production and quality of rape seed in China and the other regions of the world [23]. The present work suggests that chaetoviridin A (**3**) showed promising bioactivity against *S. sclerotiorum*. Thus, natural products—especially those from *C. globosum* species—remain a diverse source of bioactive lead molecules for both agricultural and pharmaceutical uses.

## Figures and Tables

**Table 1:** NMR spectroscopic data of **1** in CDCl_3_.

Position	δ_C_	δ_H_
1	166.2	-
3	151.8	-
4	104.3	6.15 (s)
5	136.8	-
6	102.0	6.29 (s)
7	161.3	-
8	110.4	-
9	161.3	-
9-OH	-	11.42 (s)
10	100.0	-
11	120.0	5.95 (dd, 0.8, 15.6)
12	142.3	6.48 (dd, 8.1, 15.6)
13	38.8	2.22 (m)
14	29.4	1.42 (m)
15	11.8	0.89 (t, 7.4)
16	19.7	1.07 (d, 6.7)
17	7.8	2.17 (s)

**Table 2:** NMR spectroscopic data of **2** in CDCl_3_.

Position	δ_C_	δ_H_
2	178.4	-
3	74.8	-
4a	130.6	-
4	124.7	7.15 (s)
5	137.4	-
6	130.1	7.05 (d, 7.8)
7	110.7	6.77 (d, 7.8)
7a	138.6	-
8	48.8	2.98 (d, 17.1)
		3.16 (d, 17.1)
9	207.9	-
10	31.6	2.17 (s)
11	34.1	3.29 (d, 7.3)
12	123.5	5.26 (t, 7.3)
13	133.0	-
14	25.9	1.74 (s)
15	17.9	1.70 (s)

**Table 3:** Inhibition rates (%) of compounds against five phytopathogenic fungi.

Pathogenic Fungi	1	2	3	4	5
*S. sclerotiorum*	39.0	21.5	97.8	3.5	0.5
*B. cinerea*	18.8	0	69.1	9.9	20.5
*F. graminearum*	24.0	7.9	77.0	1.6	2.6
*P. capsici*	13.7	8.5	60.7	6.6	8.6
*F. moniliforme*	31.6	6.3	59.2	12.5	15.7

**Table 4:** In vivo efficacy of compounds on cole leaves infected by *S. sclerotiorum* ^1.^

Compound	Treatment (μg/mL)	Diameter Lesion Length (mm)	Protective Efficacy (%)
**3**	200	12.5 ± 0.9	64.3
	100	16.5 ± 1.2	45.2
Carbendazim ^2^	200	11.5 ± 0.6	69.2
	100	16.6 ± 0.5	44.6
Negative control	-	26.0 ± 1.4	-

^1^ Values are the average of 5 replicates. ^2^ Positive control.

## Supplementary Materials

The following are available online, NMR spectra of compounds **1** and **2**.

## References

[1] Zhang G.Z., Zhang Y.H., Qin J.C., Qu X.Y., Liu J.L., Li X., Pan H.Y. (2013). Antifungal metabolites produced by *Chaetomium globosum* No.04, an endophytic fungus isolated from *Ginkgo biloba*. Indian J. Microbiol..

[2] Cantrell C.L., Dayan F.E., Duke S.O. (2012). Natural products as sources for new pesticides. J. Nat. Prod..

[3] Fatima N., Muhammad S.A., Khan I., Qazi M.A., Shahzadi I., Mumtaz A., Hashmi M.A., Khan A.K., Ismail T. (2016). *Chaetomium* endophytes: A repository of pharmacologically active metabolites. Acta Physiol. Plant..

[4] Yan W., Li S.J., Guo Z.K., Zhang W.J., Wei W., Tan R.X., Jiao R.H. (2017). New *p*-terphenyls from the endophytic fungus *Aspergillus* sp. YXf3. Bioorg. Med. Chem. Lett..

[5] Li L.M., Zou Q., Li G.Y. (2010). Chromones from an ascomycete, *Chaetomium aureus*. Chin. Chem. Lett..

[6] Li H., Liao Z.B., Tang D., Han W.B., Zhang Q., Gao J.M. (2018). Polyketides from two Chaetomium species and their biological functions. J. Antibiot..

[7] Chen C.M., Wang J., Zhu H.C., Wang J.P., Xue Y.B., Wei G.Z., Guo Y., Tan D.D., Zhang J.W., Yin C.P. (2016). Chaephilones A and B, two new azaphilone derivatives isolated from *Chaetomium globosum*. Chem. Biodivers..

[8] Chen C.M., Tong Q.Y., Zhu H.C., Tan D.D., Zhang J.W., Xue Y.B., Yao G.M., Luo Z.W., Wang J.P., Wang Y.Y. (2016). Nine new cytochalasan alkaloids from *Chaetomium globosum* TW1-1 (Ascomycota, Sordariales). Sci. Rep..

[9] Youn U.J., Sripisut T., Park E.J., Kondratyuk T.P., Fatima N., Simmons C.J., Wall M.M., Sun D., Pezzuto J.M., Chang L.C. (2015). Determination of the absolute configuration of chaetoviridins and other bioactive azaphilones from the endophytic fungus *Chaetomium globosum*. Bioorg. Med. Chem. Lett..

[10] Qin J.C., Gao J.M., Zhang Y.M., Yang S.X., Bai M.S., Ma Y.T., Laatsch H. (2009). Polyhydroxylated steroids from an endophytic fungus, *Chaetomium globosum* ZY-22 isolated from *Ginkgo biloba*. Steroids.

[11] Ge H.M., Yan W., Guo Z.K., Luo Q., Feng R., Zang L.Y., Shen Y., Jiao R.H., Xu Q., Tan R.X. (2011). Precursor-directed fungal generation of novel halogenated chaetoglobosins with more preferable immunosuppressive action. Chem. Commun..

[12] Yan W., Ge H.M., Wang G., Jiang N., Mei Y.N., Jiang R., Li S.J., Chen C.J., Jiao R.H., Xu Q. (2014). Pictet–Spengler reaction-based biosynthetic machinery in fungi. Proc. Natl. Acad. Sci. USA.

[13] Xiao Y., Li H.X., Li C., Wang J.X., Li J., Wang M.H., Ye Y.H. (2013). Antifungal screening of endophytic fungi from *Ginkgo biloba* for discovery of potent anti-phytopathogenic fungicides. FEMS Microbiol. Lett..

[14] Ye Y.H., Xiao Y., Ma L., Li H.X., Xie Z.L., Wang M.H., Ma H.T., Tang H.W., Liu J.Y. (2013). Flavipin in *Chaetomium globosum* CDW7, an endophytic fungus from *Ginkgo biloba*, contributes to antioxidant activity. Appl. Microbiol. Biotechnol..

[15] Zhao S.S., Zhang Y.Y., Yan W., Cao L.L., Xiao Y., Ye Y.H. (2017). *Chaetomium globosum* CDW7, a potential biological control strain and its antifungal metabolites. FEMS Microbiol. Lett..

[16] Takahashi M., Koyama K., Natori S. (1990). Four new azaphilones from *Chaetomium globosum* var. *flavor-viridae*. Chem. Pharm. Bull..

[17] Jiao W.X., Feng Y.J., Blunt J.W., Cole A.L.J., Munro M.H.G. (2004). Chaetoglobosins Q, R, and T, three further new metabolites from *Chaetomium globosum*. J. Nat. Prod..

[18] Awad N.E., Kassem H.A., Hamed M.A., El-Naggar M.A.A., El-Feky A.M.M. (2014). Bioassays guided isolation of compounds from *Chaetomium globosum*. J. Mycol. Med..

[19] Park J.H., Choi G.J., Jang K.S., Lim H.K., Kim H.T., Cho K.Y., Kim J.C. (2005). Antifungal activity against plant pathogenic fungi of chaetoviridins isolated from *Chaetomium globosum*. FEMS Microbiol. Lett..

[20] Wang W.Y., Liao Y.Y., Chen R.X., Hou Y.P., Ke W.Q., Zhang B.B., Gao M.L., Shao Z.Z., Chen J.M., Li F. (2018). Chlorinated azaphilone pigments with antimicrobial and cytotoxic activities isolated from the deep sea derived fungus *Chaetomium* sp. NA-S01-R1. Mar. Drugs.

[21] Kamano Y., Zhang H.P., Ichihara Y., Kizu H., Komiyama K., Pettit G.R. (1995). Convolutamydine-A, a novel bioactive hydroxyoxindole alkaloid from marine bryozoan *Amathia-convoluta*. Tetrahedron Lett..

[22] Nakamura T., Shirokawa S.I., Hosokawa S., Nakazaki A., Kobayashi S. (2006). Enantioselective total synthesis of convolutamydines B and E. Org. Lett..

[23] Wang Y., Hou Y.P., Chen C.J., Zhou M.G. (2014). Detection of resistance in *Sclerotinia sclerotiorum* to carbendazim and dimethachlon in Jiangsu Province of China. Australas. Plant Pathol..

