# Supplementary material for: New Metabolites from Endophytic Fungus Chaetomium globosum CDW7
Source: Molecules. 2018 Nov 4;23(11):2873. doi: 10.3390/molecules23112873 (PMC6278401; doi:10.3390/molecules23112873)
Supplement: Supplementary file 1 [file molecules-23-02873-s001.pdf]

SUPPORTING INFORMATION

New metabolites from endophytic fungus *Chaetomium globosum* CDW7

Wei Yan [1, 2,†], Ling-Ling Cao [1, 2,†], Yang-Yang Zhang [1, 2], Ran Zhao [1, 2], Shuang-Shuang Zhao [1, 2], Babar Khan [1, 2], Yong-Hao Ye [1, 2, *]

[1] State & Local Joint Engineering Research Center of Green Pesticide Invention and Application, College of Plant Protection, Nanjing Agricultural University, Nanjing 210095, China;

[2] Key Laboratory of Integrated Management of Crop Diseases and Pests, Ministry of Education, Nanjing 210095, China

[*] Corresponding Author: Email: yeyh@njau.edu.cn; Phone: +86-25-8439-9753; Fax: +86-25-8439-9753.

[†] These two authors contributed to this work equally.

Table of Content

For compound **1**:
S1. ^{1}H NMR spectrum (600 MHz) of **1** in $CDCl_3$
S2. ^{13}C NMR spectrum (150 MHz) of **1** in $CDCl_3$
S3. ^{1}H–^{1}H COSY spectrum of **1** in $CDCl_3$
S4. HSQC spectrum of **1** in $CDCl_3$
S5. HMBC spectrum of **1** in $CDCl_3$
S6. ROESY spectrum of **1** in $CDCl_3$。

For compound **2**:
S7. ^{1}H NMR spectrum (600 MHz) of **2** in $CDCl_3$
S8. ^{13}C NMR spectrum (150 MHz) of **2** in $CDCl_3$
S9. ^{1}H–^{1}H COSY spectrum of **2** in $CDCl_3$
S10. HSQC spectrum of **2** in $CDCl_3$
S11. HMBC spectrum of **2** in $CDCl_3$
S12. ROESY spectrum of **2** in $CDCl_3$。

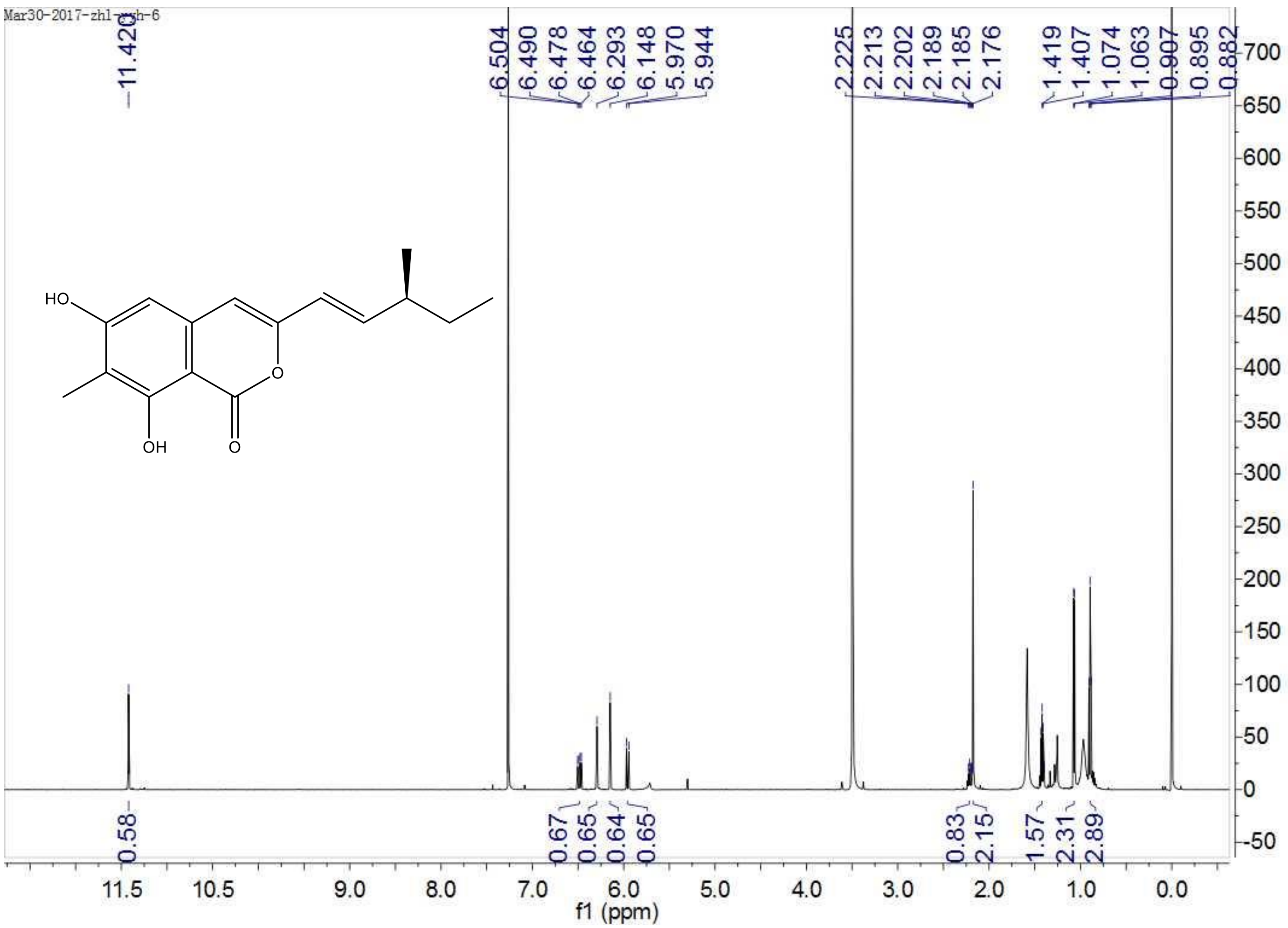

S1. The ^{1}H NMR spectrum of **1** ($CDCl_3$, 600 MHz)

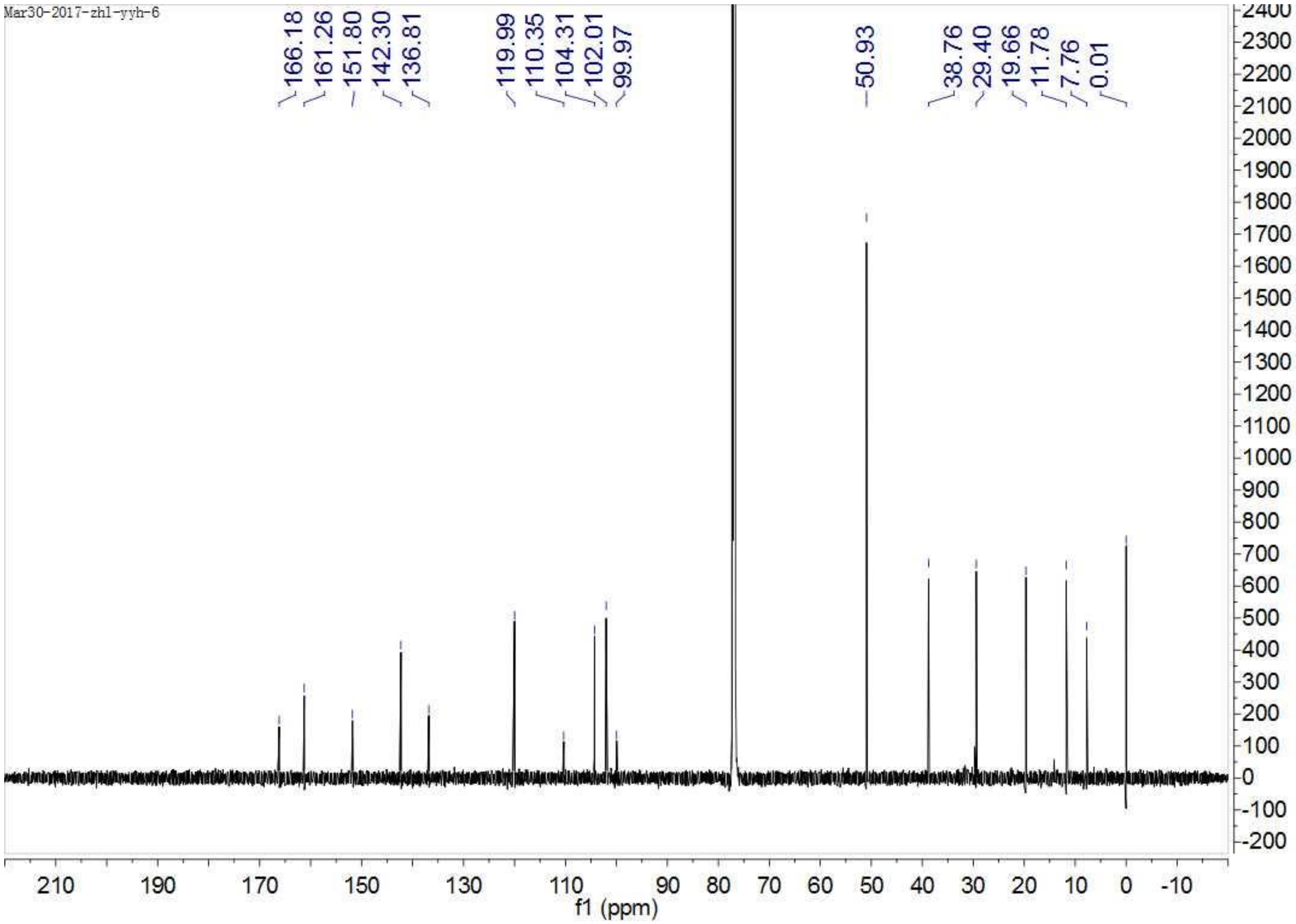

S2. The ^{13}C NMR spectrum of **1** ($CDCl_3$, 150 MHz)

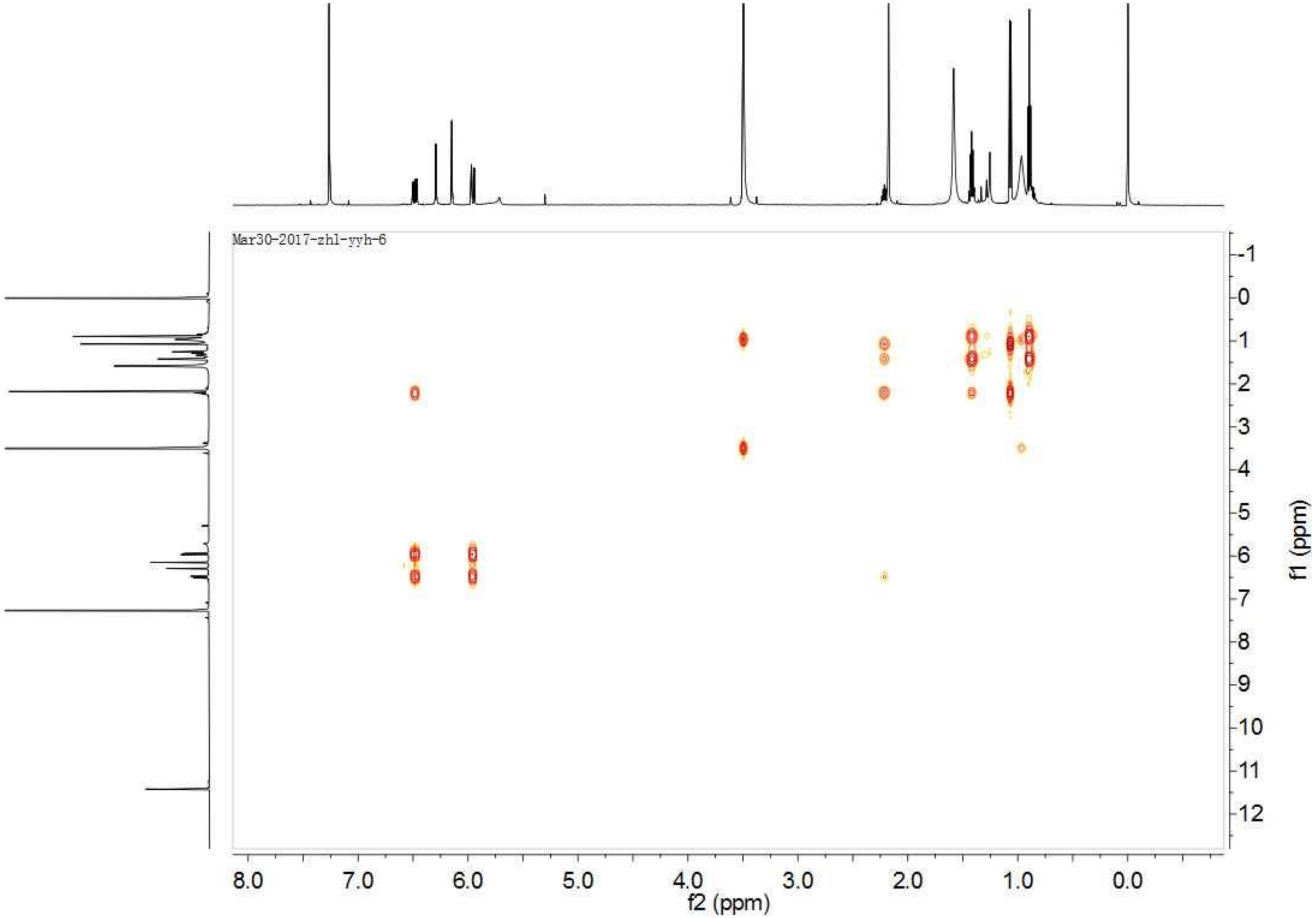

S3. The ^{1}H–^{1}H COSY spectrum of **1** ($CDCl_3$, 600 MHz)

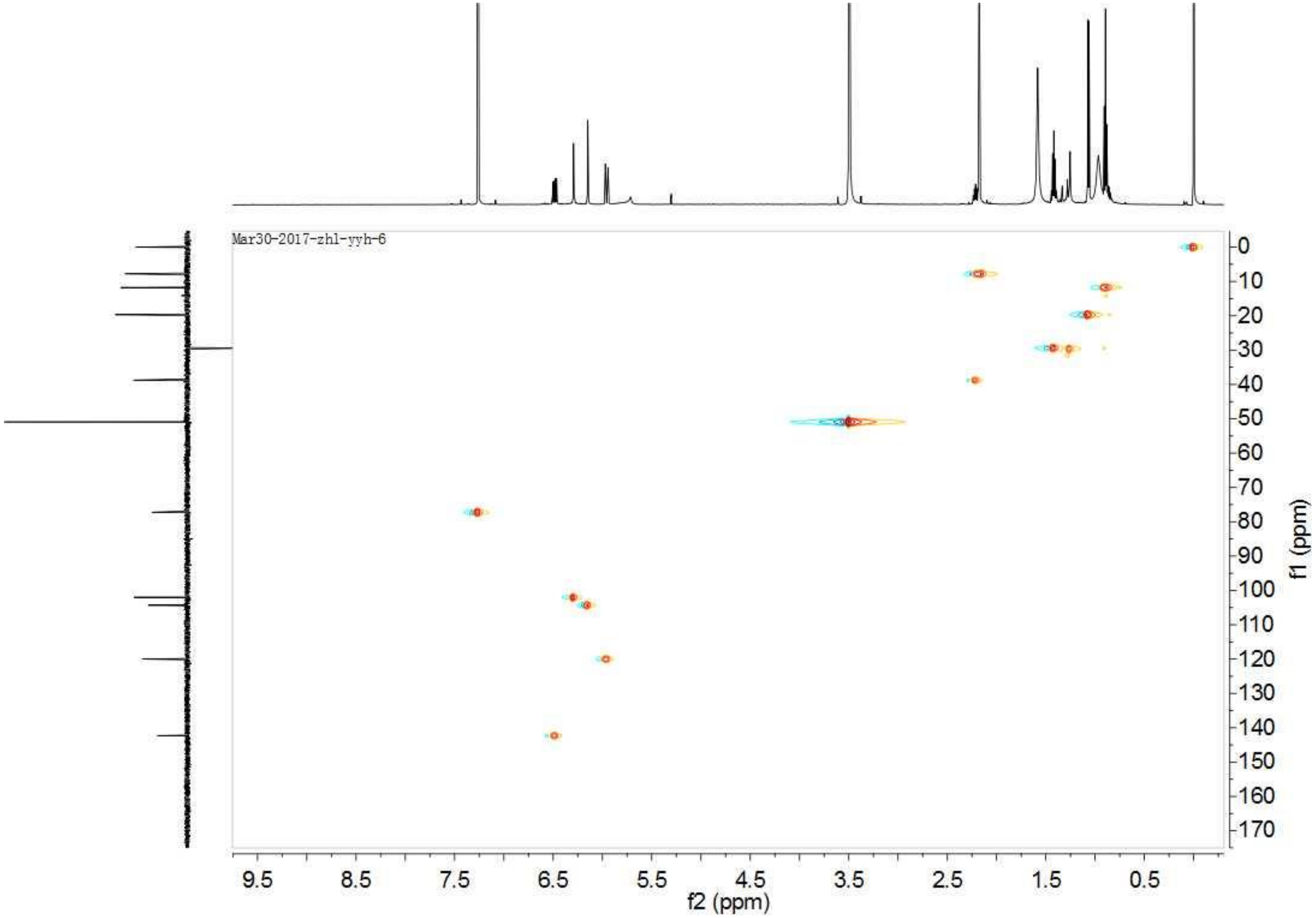

S4. The HMQC spectrum of **1** ($CDCl_3$, 600 MHz)

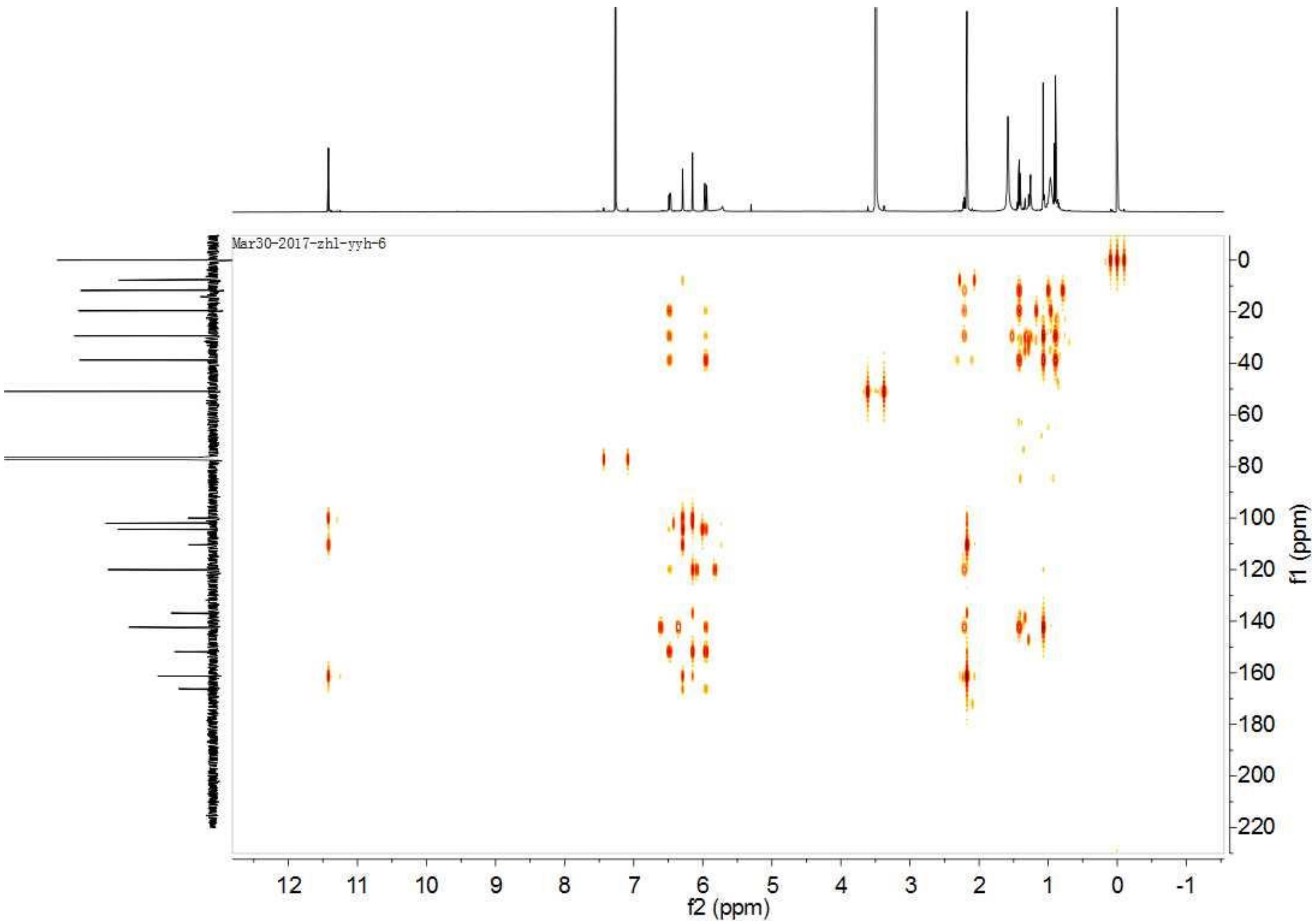

S5. The HMBC spectrum of **1** ($CDCl_3$, 600 MHz)

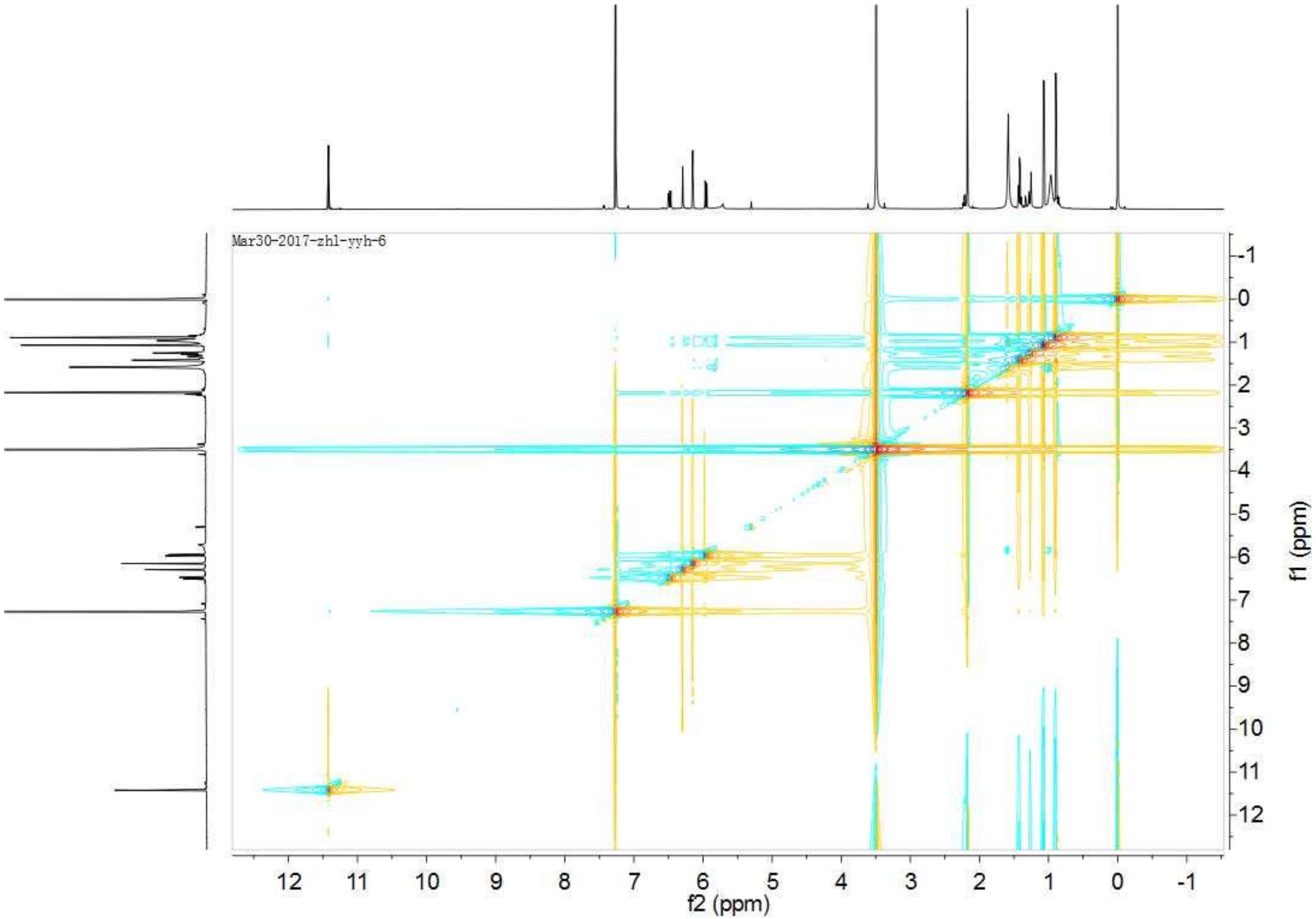

S6. The ROESY spectrum of **1** ($CDCl_3$, 600 MHz)

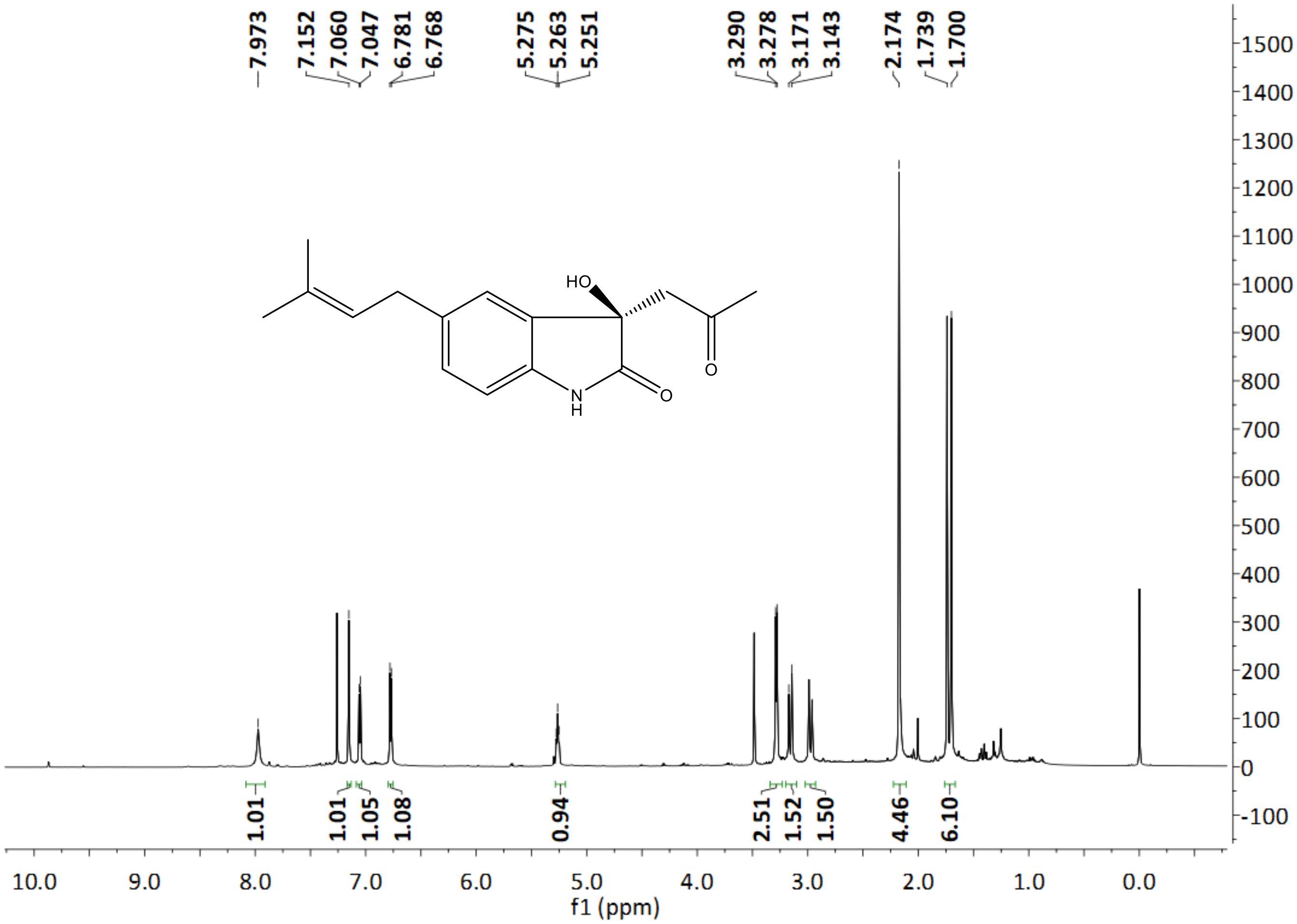

S7. The ^{1}H NMR spectrum of **2** ($CDCl_3$, 600 MHz)

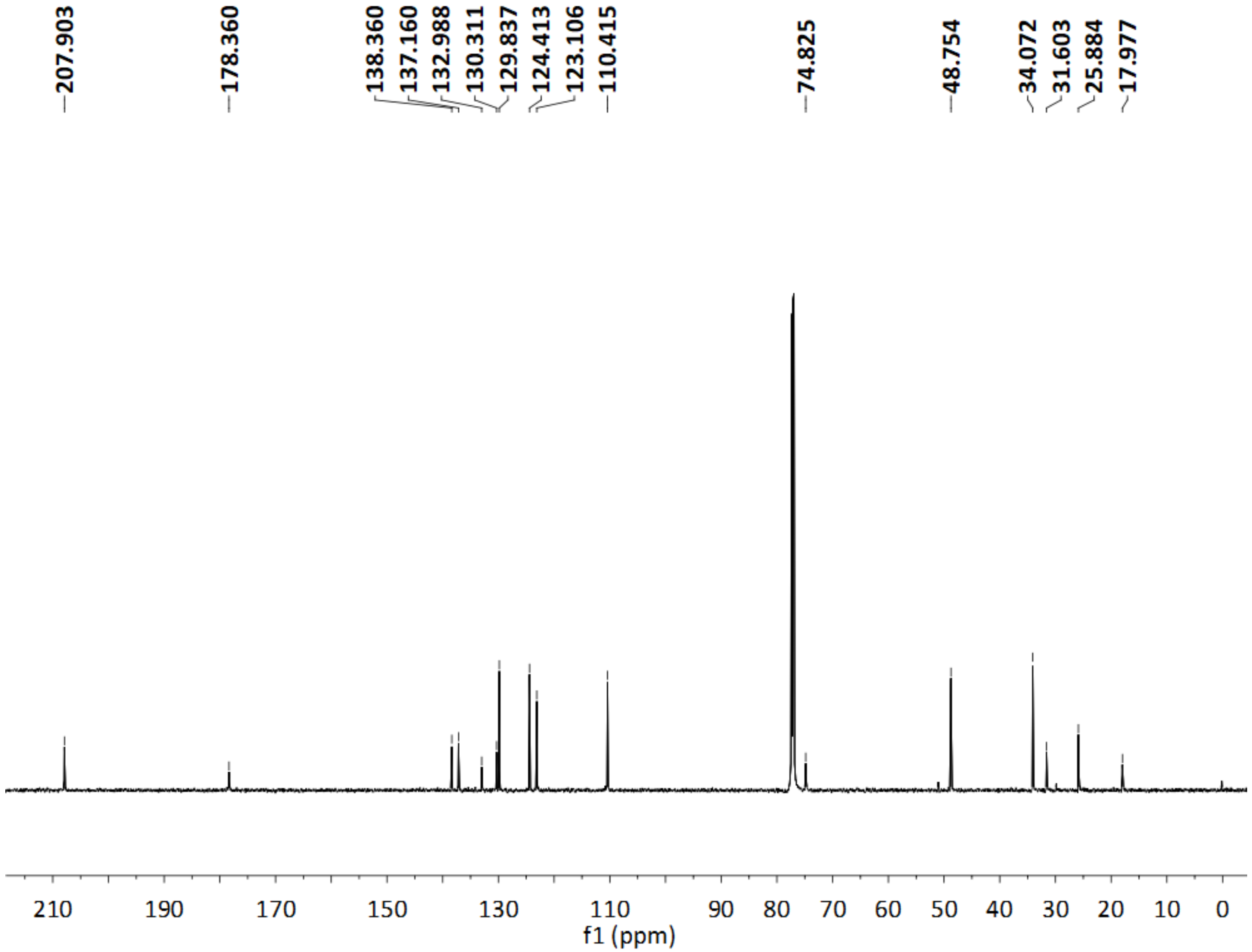

S8. The ^{13}C NMR spectrum of **2** ($CDCl_3$, 150 MHz)

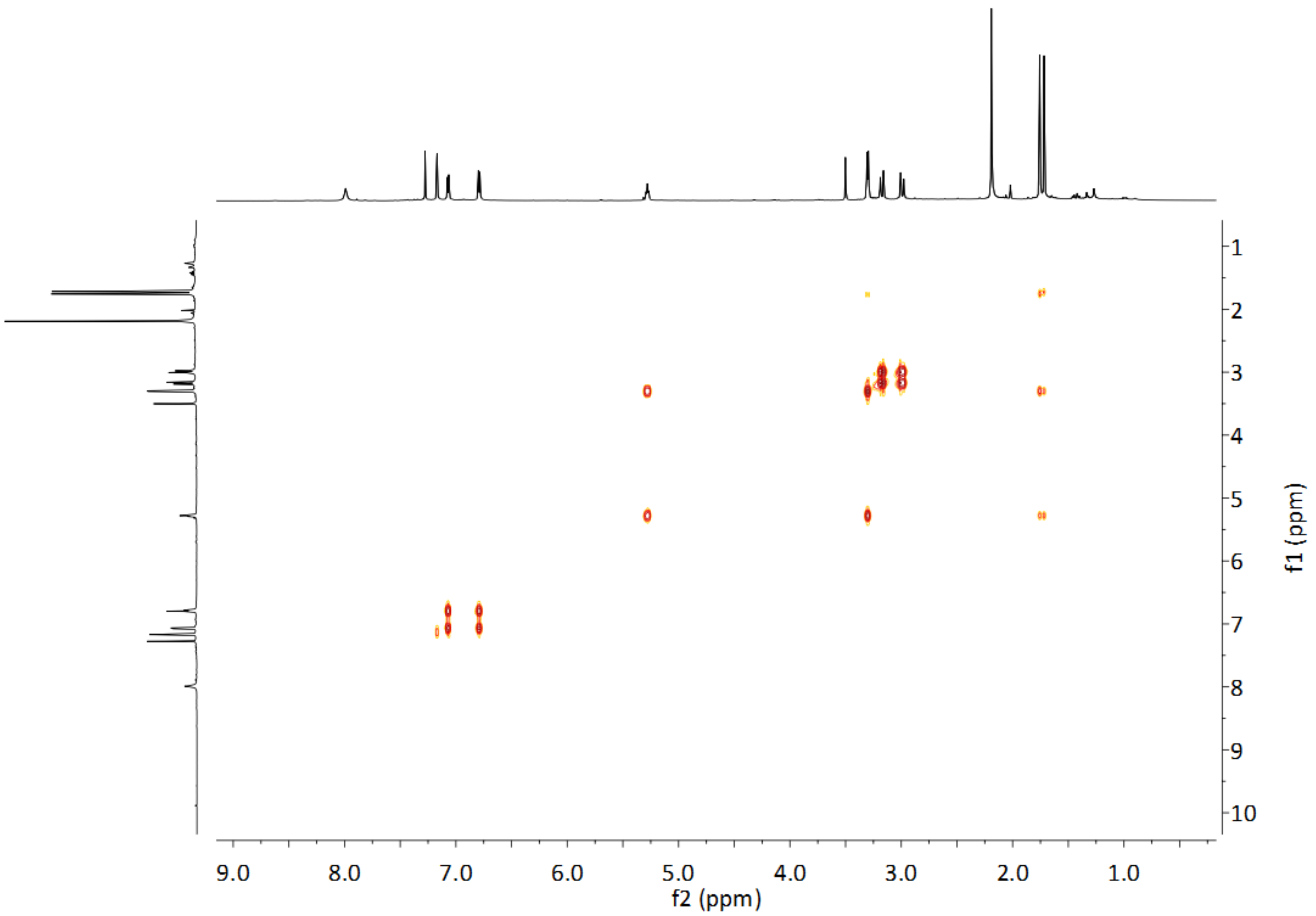

S9. The ^{1}H–^{1}H COSY spectrum of **2** ($CDCl_3$, 600 MHz)

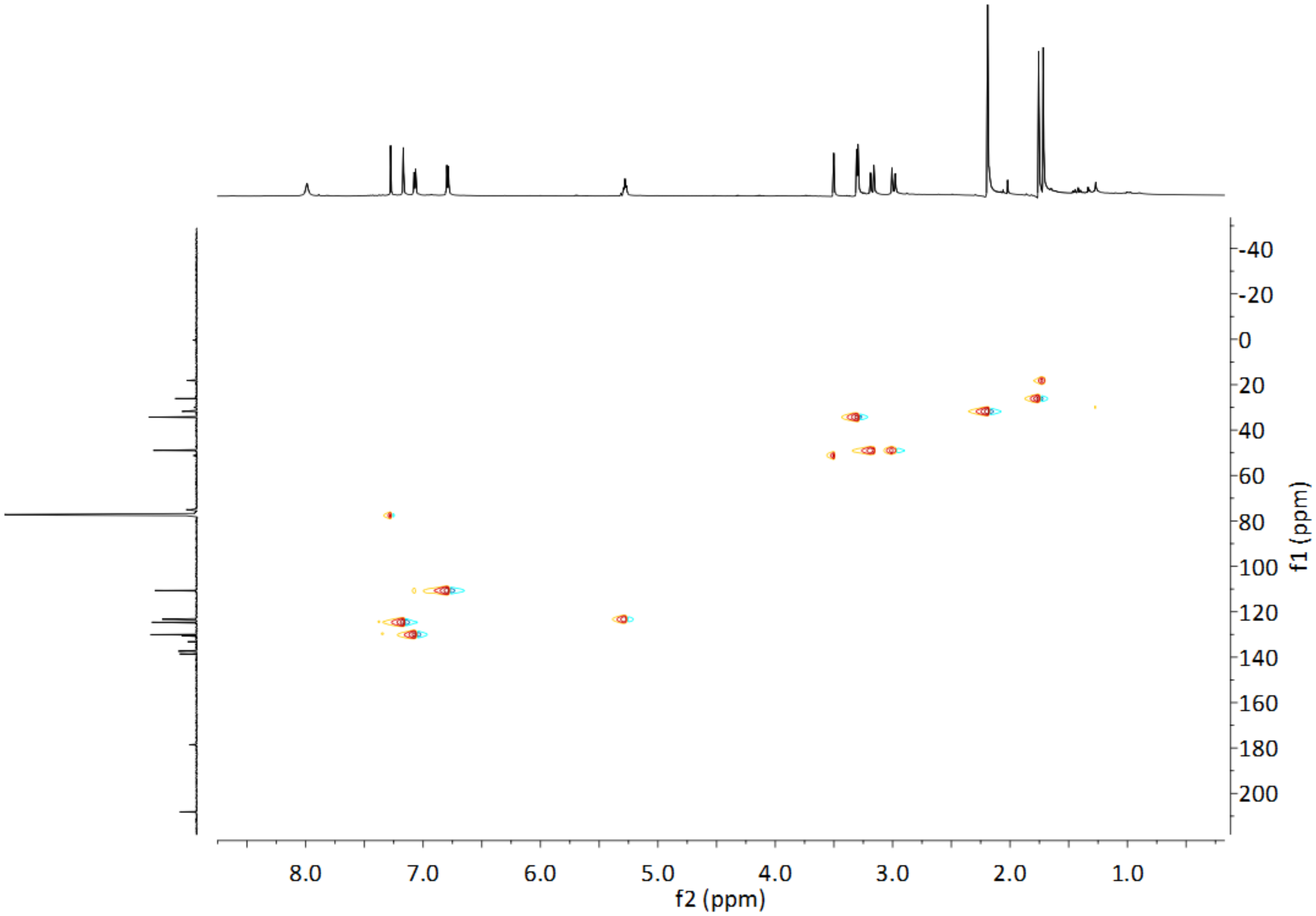

S10. The HMQC spectrum of **2** ($CDCl_3$, 600 MHz)

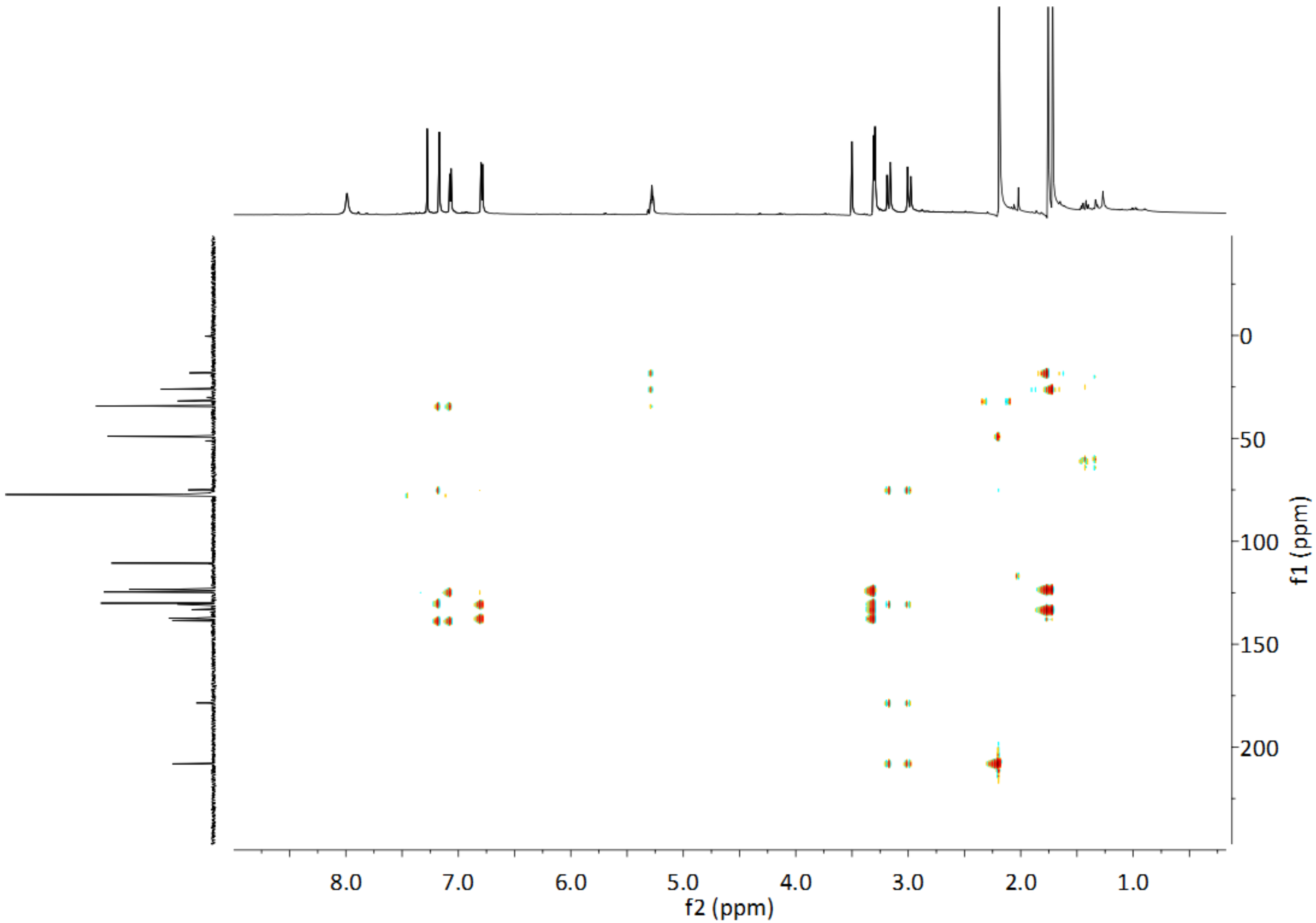

S11. The HMBC spectrum of **2** ($CDCl_3$, 600 MHz)

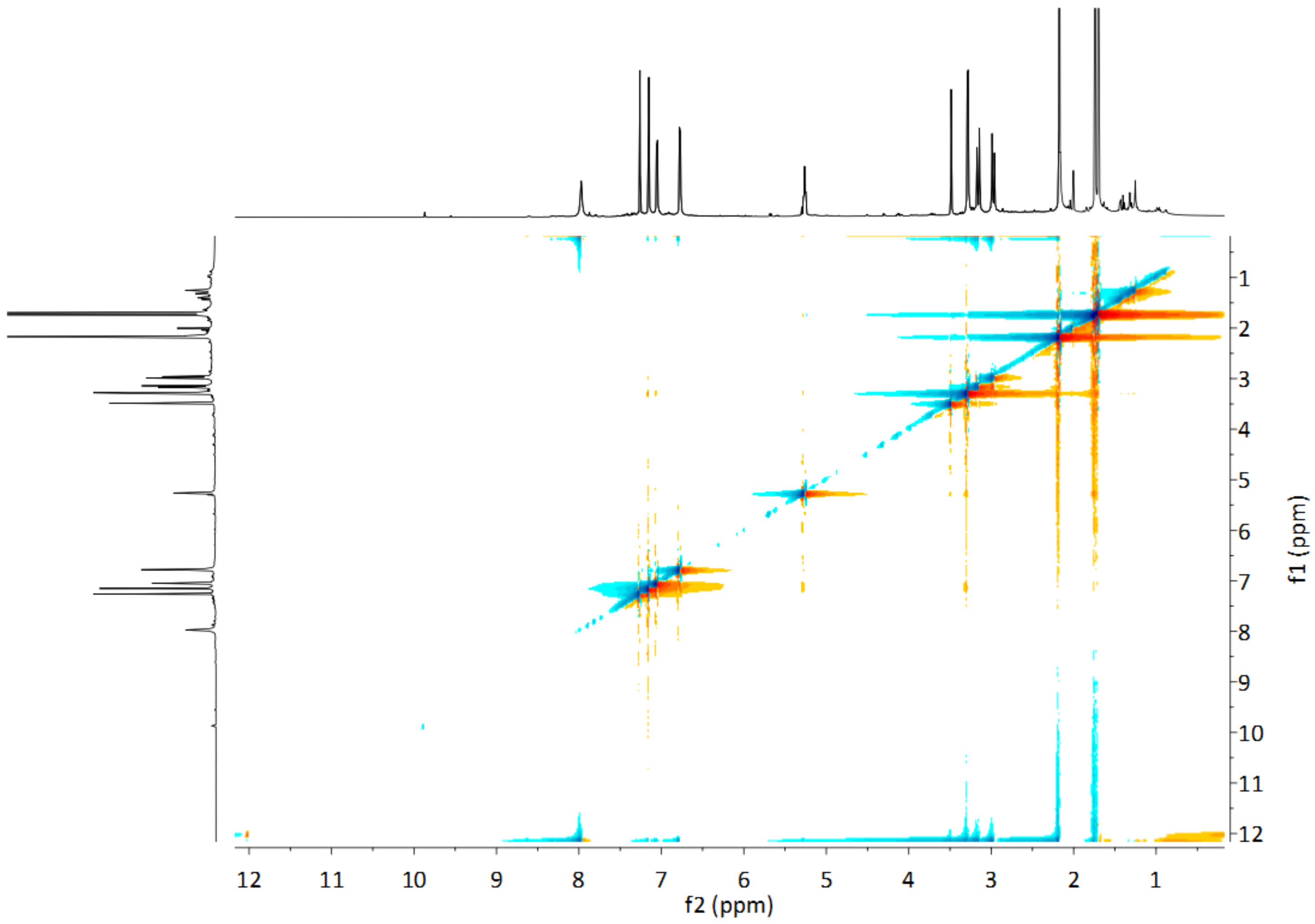

S12. The ROESY spectrum of **2** ($CDCl_3$, 600 MHz)
